# Feasibility of a school-based physical activity intervention for adolescents with disability

**DOI:** 10.1186/s40814-021-00857-5

**Published:** 2021-06-04

**Authors:** Angus A. Leahy, Sarah G. Kennedy, Jordan J. Smith, Narelle Eather, James Boyer, Matthew Thomas, Nora Shields, Ben Dascombe, David R. Lubans

**Affiliations:** 1 Priority Research Centre for Physical Activity and Nutrition, Faculty of Education and Arts, School of Education, University of Newcastle, Callaghan, New South Wales Australia; 2 New South Wales Department of Education, Sydney, New South Wales Australia; 3 Department of Physiotherapy, Podiatry and Prosthetics and Orthotics, La Trobe University, Melbourne, Victoria Australia; 4 Applied Sport Science and Exercise Testing Laboratory, School of Life and Environmental Sciences, University of Newcastle, Ourimbah, New South Wales Australia

**Keywords:** Exercise, Disability, Feasibility, School, Intervention, HIIT

## Abstract

**Background:**

Adolescents with disability are less active and have lower levels of physical fitness than their typically developing peers. Schools are ideal settings to address this; however, few school-based interventions have been designed and evaluated among this group. Therefore, the aim of this pilot study was to determine the feasibility of a time-efficient school-based physical activity intervention for adolescents with disability.

**Methods:**

A non-randomized pilot trial was conducted with adolescents in the special education unit at one secondary school in New South Wales, Australia. Sixteen grade 11 and 12 students (aged 17.3 ± 0.7 years) participated in the 2-month physical activity intervention. Two classroom teachers were trained to facilitate the delivery of a high-intensity interval training (HIIT) program, known as Burn 2 Learn adapted (B2La). Teachers were asked to deliver 2–3 weekly HIIT sessions for a period of 2 months. Four domains of feasibility (acceptability, implementation, adaptability, and practicality) were assessed using quantitative measures at the student and teacher levels (e.g., observations, process evaluation questionnaires, and heart rate [HR] monitoring). Data were also collected from three learning and support teachers who assisted classroom teachers with intervention delivery. Preliminary efficacy of the intervention on measures of adolescents’ functional capacity (6-min walk/run test) and muscular fitness (sit-to-stand test and modified push-up test) were analyzed using paired sample t-tests.

**Results:**

Moderate-to-high levels of program satisfaction were reported by both students (80% rated “Good” or “Excellent”) and teachers (100% rated “Good” or “Excellent”). Teachers reported delivering 2.5 ± 0.7 sessions per week during the study. Based on researcher session observations, the program was delivered effectively by teachers (14/20). However, HR data indicated session intensity was lower than intended. The program was considered “adaptable” by teachers, with several observed modifications to HIIT sessions to cater for the needs of adolescents with disability. No adverse events were reported. We observed improvements in preliminary efficacy measures.

**Conclusions:**

Our findings suggest it is feasible to train teachers to deliver a school-based HIIT program for adolescents with disability. Evaluation of B2La within a larger-scale effectiveness trial is warranted.

**Trial registration:**

ACTRN12621000219886.

## Key messages regarding feasibility

1. What uncertainties existed regarding the feasibility?
  There is a need to identify innovative and time-efficient school-based physical activity interventions targeting older adolescents with disability.
  High-intensity interval training (HIIT) may be one such approach; however, the feasibility of delivering school-based HIIT programs with individuals with disability is relatively unknown.
2. What are the key feasibility findings?
  The Burn 2 Learn adapted (B2La) intervention was well received by both students and teachers, contributing to effective program implementation.
  B2La was considered highly adaptable and easy to implement in the school setting with limited researcher involvement, therefore increasing the potential for intervention sustainability.
3. What are the implications of the feasibility findings for the design of the main study?
  Findings from the study will be used to refine B2La before progressing to a large-scale effectiveness trial.

## Introduction

Disability is an umbrella term used to describe impairments (i.e., problems in body function or structure), activity limitations (i.e., difficulty in performing activities), and participation restrictions (i.e., problems engaging in life situations) [1]. Participating in regular physical activity is associated with a plethora of health benefits in adolescents (aged 10–19 years), including higher levels of physical fitness [2], and is especially beneficial for adolescents with disability [3]. Disability is recognized as a global public health issue, as people with disability face barriers in accessing health and related services, often resulting in poor health [4]. As noted in the World Health Organization’s global disability action plan 2014–2021 [5], the burden of disability can be reduced by addressing physical activity participation. Unfortunately, adolescents with disability have been largely neglected in health promotion efforts. Physical inactivity is a serious health concern among the general public, but rates of inactivity are even higher among individuals with disability [6]. Moreover, adolescents with disability have poorer physical fitness than their peers without disability [7].

Adolescents with disability face many common and unique barriers to participation in physical activity. Previous research has identified personal (i.e., lack of skills and time to exercise), social (i.e., unsupportive peers and parents), and environmental (i.e., inadequate accessibility and lack of appropriate programs) barriers to participation [8]. Conversely, factors that may facilitate participation include having the time available, being involved in programs that are flexible/adaptable, and exercising in a group with people of a similar age [9]. It is important to acknowledge that parents play a crucial role in determining whether adolescents with disability are physically active. While parents acknowledge the value of physical activity and want their children to be active, they have also expressed their concern regarding extensive time commitments (i.e., travel time), balancing the need of family members, and program suitability as barriers to participation [10]. The delivery of physical activity programs in the school setting may help mitigate these barriers. To date, the majority of physical activity interventions targeting young people with disability have been conducted in clinical, community, and home settings [11].

Schools are ideal settings for physical activity promotion, as they provide access to the majority of the pediatric population and have the necessary equipment, facilities, and personnel to deliver programs [12]. Physical education (PE) is the primary means of physical activity promotion in schools, and there is a growing body of research focusing on the inclusion of adolescents with disability in inclusive PE classes [13]. While teachers typically advocate for inclusion in PE, many teachers lack the confidence and competence to successfully integrate students with disability [14]. Regardless, simply integrating students with disability into inclusive PE may not be enough to produce meaningful changes in health. As such, identifying strategies that allow for regular physical activity opportunities during the school day are warranted. In general, students have fewer opportunities to be physically active during their final school years, when time is typically re-allocated toward other traditional academic subjects [15].

Lack of time has emerged as one of the most common barriers to physical activity promotion in schools [16]. As such, there is a need for school-based physical activity interventions for students with disability that are also time-efficient. High-intensity interval training (HIIT) is a time-efficient mode of exercise that can improve physical, mental, and cognitive health in typically developing children and adolescents [17, 18]. School-based HIIT interventions are becoming increasingly popular; however, process data gathered from those directly involved in the interventions (i.e., students and teachers) are lacking [19, 20]. To our knowledge, only two previous studies have evaluated school-based HIIT programs for adolescents with disability [21, 22]. While both studies found HIIT was effective in improving physical health measures (i.e., body composition, aerobic fitness), they did not report detailed feasibility findings. Such information is needed to inform the design of future school-based HIIT studies. Therefore, the primary aim of this study is to determine the feasibility of a school-based physical activity intervention for adolescents with disability.

## Methods

### Study design, participants, and setting

A non-randomized pilot trial was conducted at one mainstream secondary school, located in New South Wales (NSW), Australia. Grade 11 and 12 students in the special education faculty were eligible to participate in the study. Two special education teachers (i.e., classroom teachers) from the same school were recruited to deliver the intervention, while three learning and support teachers were also involved in assisting intervention delivery. The study school caters to students in grades 11 and 12 (i.e., senior campus), with approximately 600 students enrolled. The school’s Index of Community Socio-Educational Advantage (ICESA) value is in the 32nd percentile for Australian schools (i.e., less advantaged than 68% of schools nationally). Ethics approval for the study was obtained from the Human Research Ethics Committee of the University of Newcastle, Australia (H-2016-0424), and the NSW Department of Education (SERAP 2017116). School principals, teachers, and parents of students provided informed written consent prior to enrolment. Students provided written assent. This study was retrospectively registered with the Australian and New Zealand Clinical Trials Registry (Trial ID: ACTRN12621000219886).

### Intervention

The intervention was an adaptation of the Burn 2 Learn (B2L) program- a school-based high-intensity interval training (HIIT) intervention designed to improve older adolescents’ physical, mental, and cognitive health [23, 24]. A typical B2L session lasts ~ 10 min and involves 8 × 30-s work intervals (≥ 85% age-predicted maximum heart rate), interspersed with 30-s rest intervals. The sessions include a combination of aerobic (e.g., shuttle runs, jumping jacks, high knees running) and resistance (e.g., push-ups, body weight squat, lunges) exercises. For the current study, an adapted version of the B2L program, hereafter referred to as B2L adapted (B2La), was implemented. Before the intervention, classroom teachers attended a half-day professional development workshop led by members of the research team. The workshop provided teachers with a rationale for B2La, with a specific focus on the impact of vigorous physical activity for students’ health. Prior to intervention delivery (i.e., during the professional learning workshop), the research team made adaptations to the original intervention (i.e., B2L) to make it more appropriate for students with disability. Specifically, exercise sessions were designed to have low complexity during the early weeks of the intervention. For example, rather than alternating between two different exercises, students repeated the same exercise for the full work interval (i.e., performing squats for 30 s). Once students were more competent at performing the exercises in isolation, teachers were advised to increase the complexity by including both aerobic and resistance exercises within the same work interval (i.e., four squats + 10-m shuttle run, repeated for 30 s). Teachers were reminded about the importance of modifying exercises to suit students’ fitness and ability levels. For example, if a student was unable to perform a standard push-up, teachers encouraged students to perform modified push-ups (i.e., on knees or against a wall). Teachers also participated in a practical HIIT session where they were familiarized with the intervention resources. Following the workshop, classroom teachers were asked to deliver 2–3 weekly B2La sessions for 2 months. Learning and support teachers assisted classroom teachers in their delivery of B2La sessions (e.g., provide demonstrations for students, help set up equipment, and encourage student participation), however were not responsible for leading the delivery of sessions, and therefore did not attend the professional development workshop.

In accordance with self-determination theory (SDT) [25], HIIT sessions were designed to satisfy students’ basic psychological needs for autonomy (e.g., providing students with choice), competence (e.g., adapting exercises to meet the needs/fitness levels of students), and relatedness (e.g., promoting an inclusive group environment). SDT components were operationalized using the SAAFE (Supportive, Active, Autonomous, Fair, Enjoyable) delivery principles [26], which were explained to teachers during the professional development workshop. Teachers were encouraged to modify HIIT sessions to cater for student skill and fitness levels. To provide real-time feedback during HIIT sessions, students were equipped with heart rate monitors (Wahoo TICKR), which paired to a purpose-built B2L iPad application displaying heart rate during HIIT sessions. Teachers were also provided with a suite of pre-designed hard-copy HIIT task and technique cards to develop students’ HIIT competence.

### Study measures

Feasibility was assessed across five domains based on the framework described by Bowen et al. [27]. Table 1 provides an overview of each domain assessed at the student and teacher level. Both students and classroom teachers completed a post-program questionnaire regarding their experiences with various aspects of the intervention. The learning and support teachers who were involved in facilitating HIIT sessions but who did not attend the professional development workshop also completed the post-program questionnaire. At baseline, standard demographic information (i.e., age, sex) were collected from students using a paper and pencil questionnaire. Health-related fitness assessments were conducted prior to the start of the intervention (June, 2020) and following the 2-month intervention (August, 2020) at the study school.

**Table 1:** Description of feasibility domains assessed in the current study

Domain	Description	Student outcomes	Teacher outcomes
**Acceptability**	The extent to which the program is considered suitable, satisfying, or attractive to program participants	• Program satisfaction • Barriers and motivation to HIIT • HIIT sustainability • Group exercise preferences	• Program satisfaction • Intervention sustainability
**Implementation**	The extent, likelihood, and manner in which the program can be fully implemented as planned	• Session adherence • Session intensity	• Sessions delivered • Session duration • Quality of session delivery
**Adaptation**	The extent to which an existing program can be adapted to fit the needs of different populations	n/a	• Qualitative description of session adaptations • Adaptation to school characteristics
**Practicality**	The extent to which the program can be delivered using existing resources or with limited resources, and without outside intervention	• Practicality of program resources	• Ease of implementation • Session timing acceptability • Adverse events
**Preliminary efficacy**	The extent to which the program works in making positive changes to muscular fitness and functional performance	• Sit-to-stand test • Push-up test • 6-min run/walk test • Mood state	n/a

*Acceptability* was assessed using multiple measures at the student and teacher level. Students’ program satisfaction, motivators and barriers to participation, and group exercise preferences were assessed on a 5-point scale (i.e., 1 = *Poor/Strongly disagree*, 2 = *Fair/Disagree*, 3 = *Average/Neutral*, 4 = *Good/Agree*, 5 = *Excellent/Strongly agree*). Teachers’ satisfaction was assessed on a 4-point scale (i.e., 1 = *Strongly disagree*, 2 = *Disagree*, 3 = *Agree*, 4 = *Strongly Agree*). Program sustainability was assessed on an 11-point matrix scale (i.e., 0 = *Highly unlikely* to 10 = *Highly likely*) for both students (participating in HIIT) and teachers (delivering HIIT).

*Implementation* was assessed using heart rate data gathered from a purpose-built B2L iPad application (i.e., session intensity), and the number of sessions reported by teachers (i.e., dose delivered). A member of the research team also observed classroom teachers’ HIIT session delivery (i.e., session quality) on two occasions (weeks 2 and 6 of the intervention), using the SAAFE observational checklist [26]. Session quality was assessed using a 4-point scale (i.e., 1 = *Strongly disagree* to 4 = *Strongly agree*) with items corresponding to the adherence (or lack thereof) of suggested strategies for satisfying each component of the SAAFE delivery principles. Teachers also reported the average length of HIIT sessions (i.e., 8 min, 12 min, or 16 min) and the minimum amount of time required to deliver a B2La HIIT session (including set-up and the time taken to transition back to the classroom).

*Adaptation* was determined in two ways. First, we conducted two HIIT session observations over the study period and examined classroom teachers’ program adaptations. Second, we asked teachers to reflect on their perceptions of program “adaptability” by responding to the following item: “I could adapt the program based on the characteristics of my students” using a four-point scale (i.e., 1 = *Strongly disagree* to 4 = *Strongly agree*).

*Practicality* was defined as teachers’ perception of their capacity to deliver the intervention in a school setting, and by describing any adverse events that occurred during the intervention period. Classroom and learning and support teachers’ perception of session duration satisfaction, ease of program implementation, and the availability of school facilities to support intervention delivery were assessed on a four-point scale (i.e., 1 = *Strongly disagree* to 4 = *Strongly agree*). Students were asked to rate how intervention resources (i.e., heart rate monitors) influenced their motivation during HIIT sessions on a five-point scale (i.e., 1 = *Strongly disagree* to 5 = *Strongly agree*).

*Preliminary efficacy* was assessed by evaluating within-group changes in functional performance and muscular fitness from baseline to post-test. Members of the research team were unable to conduct assessments with students due to COVID-19 restrictions. Therefore, assessments were administered by one of the trained classroom teachers, under the guidance of a member of the research team. Functional performance was assessed using a 6-min run/walk test, which has good reliability in adolescents with intellectual disability (ICC = 0.82) [28]. Students were fitted with a GPS device (GameTraka SPT 2) and instructed to cover as much distance as possible in 6 min. Lower body muscular endurance was assessed using a 30-s sit-to-stand test. From a seated position, students were required to stand up and sit back down on a regular classroom chair as many times as possible in 30 s [21]. Upper body muscular endurance was assessed using a modified push-up test [29]. All students were instructed to perform as many push-ups as possible on their knees. An exception was made for one student who was physically unable to perform push-ups on their knees (performed on toes instead). After discussion with classroom teachers, we adapted the Feeling State questionnaire for administration before and following HIIT sessions to assess students’ mood [30]. Data were collected for the first 5 weeks of the intervention (total 12 sessions). Students were asked to respond to the question “How do you feel right now?” Responses were scored on a 3-point scale (i.e., 1 = Sad, 2 = Neutral, 3 = Happy). Mean pre- and post-session scores were calculated.

### Statistical analyses

Data for acceptability, adaptation, implementation, and practicality were analyzed using descriptive statistics (i.e., mean, standard deviation, percentages), or qualitatively described where appropriate. Outcomes for preliminary efficacy were analyzed using paired sample t-tests to determine change in mean from baseline to post-test. Analyses were conducted in IBM SPSS Statistics for Windows (Version 26; 2010 SPSS Inc., IBM Company, Armonk, NY). Cohen’s *d* was calculated to provide a measure of effect by dividing the mean difference in change (post-test minus baseline) by the standard deviation of change. Consistent with Cohen’s interpretation, values of *d* = 0.2, *d* = 0.5, and *d* = 0.8 were considered as small, medium, and large effect sizes, respectively [31].

## Results

**Fig. 1:**
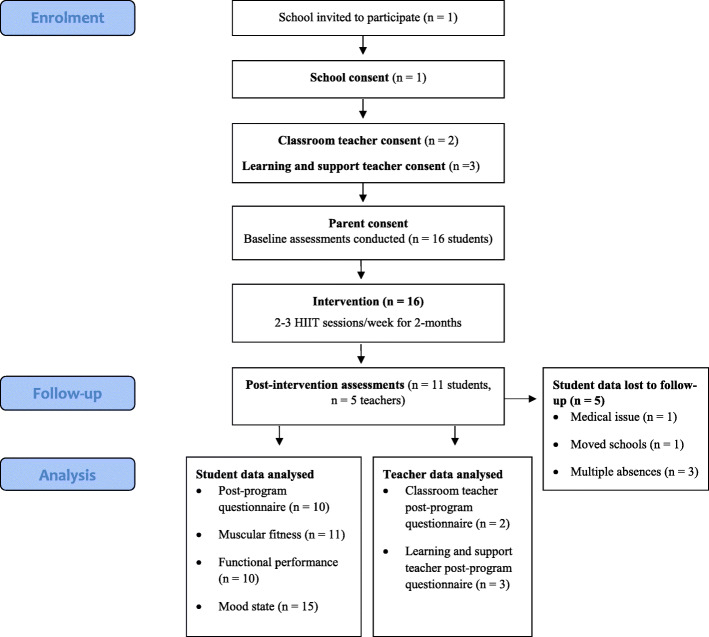
Flow of participants through the study

Figure 1 shows the flow of participants through the study. Students’ baseline characteristics are presented in Table 2. Sixteen students (11 males, 5 females, mean age 17.3 ± 0.7 years), were recruited for the study, 11 of whom completed post-intervention assessments (7 males, 4 females, mean age 17.3 ± 0.7 years). Reasons for students not completing the study were medical issue (n = 1), moving schools (n = 1), and not being available to complete follow up assessments (n = 3). Seven (64%) students with intellectual disability, three (27%) with autism, and one (9%) with a mental health disability completed the study. A total of 15 students provided pre- and post-session mood data. Feasibility data pertaining to acceptability, implementation, adaptability, and practicality outcomes are presented as mean values in Table 3.

**Table 2:** Descriptive characteristics of study participants

Characteristics	Total (N = 11)
**Age, mean (SD), years**	17.3 (0.7)
**Sex, n (%)**	
Male	7 (63.6)
Female	4 (36.4)
**Cultural background, n (%)** ^**a**^	
Australian	7 (77.8)
European	1 (11.1)
Middle Eastern	1 (11.1)
**Language spoken at home, n (%)** ^**b**^	
English	9 (90.0)
Other	1 (10.0)
**Primary diagnosis, n (%)**	
Intellectual disability	7 (63.6)
Autism	3 (27.3)
Mental health	1 (9.1)

^a^Two participants did not provide data for cultural background

^b^One participant did not provide data for language spoken at home

**Table 3:** Summary of feasibility evaluation

**Acceptability**	
**Student**	
**Satisfaction /5**	
Overall satisfaction, mean (SD)	3.9 (1.2)
Satisfaction with HIIT sessions, mean (SD)	4.1 (0.9)
Satisfaction with intervention resources, mean (SD)	4.2 (0.9)
**Motivation to participate in HIIT /5**	
Health, mean (SD)	4.2 (1.0)
Fitness and sports performance, mean (SD)	4.1 (1.2)
Mental health and well-being, mean (SD)	3.7 (1.4)
Academic performance, mean (SD)	3.4 (1.3)
Physical appearance, mean (SD)	3.8 (1.2)
**Barriers to participation in HIIT /5**	Top 3 highest ranked
Do not like to exercise, mean (SD)	2.7 (1.6)
Did not want to get sweaty, mean (SD)	2.6 (1.7)
Did not enjoy the exercises, mean (SD)	2.5 (1.5)
**Group exercise preference /5**	
Enjoyed exercising with students from the special education faculty, mean (SD)	4.4 (0.5)
Prefer to exercise with students from mainstream classes, mean (SD)	2.3 (1.3)
**Sustainability**	
Participation in future HIIT, yes, %	68.9
**Teacher**	
**Satisfaction /4**	
Classroom teacher overall satisfaction, mean (SD)	3.5 (0.7)
Support teacher overall satisfaction, mean (SD)	4.0 (0.0)
Classroom teacher satisfaction of intervention resources, mean (SD)	2.5 (0.7)
Support teacher satisfaction of intervention resources, mean (SD)	4.0 (0.0)
**Sustainability**^**a**^	
Delivery of intervention to future student cohorts, yes, %	85
**Implementation**	
**Student**	
**Session intensity**	
Average HR during session, mean beats/min (SD)	146.0 (17.3)
Average HR during sessions, mean % of HR_max_ (SD)	72 (8)
Peak HR during session, mean beats/min (SD)	164 (17)
Peak HR during sessions, mean % of HR_max_ (SD)	81 (9)
**Teacher**^**a**^	
**Session delivery**	
Classroom teacher reported sessions, mean (SD)	2.5 (0.7)
Classroom teacher participation, mean (SD)	2.5 (0.7)
Support teacher participation, mean (SD)	3.0 (0.0)
**Average HIIT session time, n (%)**^**c**^	
< 15 min	0 (0.0)
15–20 min	0 (0.0)
20–25 min	2 (40.0)
> 25 min	3 (60.0)
**Session quality**^**b**^	
Adherence to SAAFE delivery principles, mean (SD)	
Supportive /4	2.8 (1.2)
Active /4	3.2 (0.2)
Autonomous /4	1.3 (0.5)
Fair /4	3.3 (0.5)
Enjoyable /4	3.3 (0.5)
SAAFE observation total /20	14 (2.8)
**Adaptation**	
Classroom teacher adaptation to school characteristics, mean (SD)	4.0 (0.0)
Support teacher adaptation to school characteristics, mean (SD)	4.0 (0.0)
**Practicality**	
**Student /5**	
Intervention resources increased motivation to work harder	4.2 (0.9)
**Teacher /4**	
Classroom teacher session duration satisfaction, mean (SD)	3.5 (0.7)
Support teacher session duration satisfaction, mean (SD)	4.0 (0.0)
Classroom teacher ease of implementation, mean (SD)	3.5 (0.0)
Support teacher ease of implementation, mean (SD)	3.7 (0.6)
Classroom teacher school facilities available to support intervention delivery, mean (SD)	3.5 (0.7)
Support teacher school facilities available to support intervention delivery, mean (SD)	4.0 (0.0)

^a^Only classroom teachers responded to this item

^b^Researcher observations were conducted with classroom teachers who attended the professional development workshop

^c^This included time taken for exercise explanations and for students to go back to the classroom

### Acceptability

Overall acceptability of the program was moderate-to-high among students, with 80% students rating the program as “Good” (n = 5/10) or “Excellent” (n = 3/10). Program satisfaction was also high among teachers (“Good” n = 1/5, “Excellent” n = 4/5). Both classroom teachers completed a workshop evaluation and “strongly agreed” that they were confident to deliver the program following the professional development workshop. In response to the following statement “I found the exercise sessions enjoyable” student responses were “Neutral” (n = 3/10), “Agree” (n = 3/10), or “Strongly agree” (n = 4/10). Students also rated program resources highly, with 80% of responses rated as “Good” (n = 3/10) or “Excellent” (n = 5/10). Teachers’ ratings of resource quality were mixed- “The program resources were well designed and high quality” (“Disagree” n = 1, “Agree” n = 1, “Strongly agree” n = 3). Health was the strongest motivator for students, while not liking exercise was found to be the biggest barrier to participation. In response to the statement “I enjoyed doing exercise with students from the special education faculty” student responses were “Neutral” (n = 2/10), “Agree” (n = 2/10), and “Strongly agree” (n = 6/10). For the statement “I would prefer to do exercise with other students in the school” student responses were “Strongly disagree” (n = 4/10), “Disagree” (n = 2/10), “Neutral” (n = 1/10), and “Agree” (n = 3/10). The majority of students (n = 8/10) indicated they would likely participate in HIIT over the next two months, with an average likelihood of 69%. Similarly, both classroom teachers reported their intention to deliver the intervention to future student cohorts, with an average likelihood of 85%.

### Implementation

Data from heart rate monitors indicated students were working toward the prescribed intensity (≥ 85% age-predicted HR_max_). The average heart rate during sessions was 72% HR_max_, while the average peak heart rate achieved by students was 81% HR_max_. Classroom teachers reported facilitating 2.5 ± 0.7 sessions per week across the intervention period. Encouragingly, both classroom and learning and support teachers actively participated in HIIT sessions with students. Overall, data from two fidelity observations indicated that HIIT sessions were being delivered as intended by classroom teachers (14.0/20). The *Active* (3.2/4), *Fair* (3.3/4), and *Enjoyable* (3.3/4) principles were most effectively implemented by classroom teachers, while the *Autonomous* principle was implemented the least effectively (1.3/4). HIIT sessions were typically 8 min in length, with the minimum amount of time required to facilitate a HIIT session (instruction delivery and time to return to the classroom) being 20 min.

### Adaptability

All five teachers responded “Strongly agree” to the following statement “I could adapt the program based on the characteristics of my students”. The research team observed additional HIIT program adaptations made by teachers during the two session observations. First, teachers adopted a higher instructor to participant ratio (i.e., ~ 1 teacher to 4 students) than originally discussed in the professional learning workshop. Second, teachers used a shorter HIIT session duration (i.e., teachers would facilitate 2 × 4-min HIIT sessions with a small break in between, as opposed to 1 × 8-min HIIT session).

### Practicality

Teachers perceived the overall duration of HIIT sessions to be an acceptable amount of time (“Agree” n = 1/5, “Strongly agree” n = 4/5), and classroom teachers found the program was easy to implement with their students (“Agree” n = 1/2, “Strongly agree” n = 1/2). No injuries or adverse events were recorded by teachers over the study period. For the following statement “Using the heart rate monitors and iPad made me work harder in the sessions” student responses were “Neutral” (n = 3/10), “Agree” (n = 2/10), and “Strongly agree” (n = 5/10).

### Preliminary efficacy

**Fig. 2:**
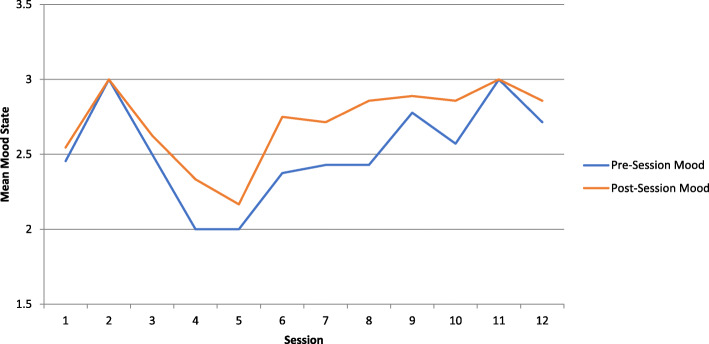
Mean mood state recorded pre- and post-HIIT session

Baseline and post-test values for health-related fitness variables are reported in Table 4. A total of 11 participants provided complete data for muscular fitness assessments. Analyses of paired samples t-tests demonstrated an increase in participants’ muscular fitness. Specifically, lower body muscular fitness performance improved from baseline to post-test (3 repetitions; 95% CI, 1 to 5, *p* = 0.013), while participants’ upper body muscular fitness performance improved following the intervention period (7 repetitions; 95% CI, 2 to 12, *p* = 0.008). Ten participants provided complete data for functional performance as assessed by the 6-min run/walk test. Improvements were found for total distance covered (163 m; 95% CI, 69.3 to 256.7, *p* = 0.003) and work rate (27.2 m/min, 95% CI, 11.5 to 42.9, *p* = 0.003). Mean pre- and post-session mood data are presented in Table 4. Mean values across the sessions are displayed in Fig. 2. Mood improved following participation in HIIT sessions (pre = 2.4 ± 0.5, post = 2.6 ± 0.4, *p* = 0.022).

**Table 4:** Changes in outcomes from baseline to post-test

Variables	N			Change in mean		
				Mean^**b**^	***p***	***d***
		**Baseline**^**a**^	**Post-test**^**a**^			
**Muscular fitness**						
Sit-to-stand repetitions	11	15 (5)	18 (5)	3 (1, 5)	0.013	0.91
Push-up repetitions	11	5 (6)	12 (12)	7 (2, 12)	0.008	0.99
**Functional performance**						
Total distance (m)	10	399.9 (126.5)	562.9 (158.3)	163 (69.3, 256.7)	0.003	1.25
Top speed (km/h)	10	15.6 (4.9)	16.8 (3.6)	1.2 (-2.0, 4.4)	0.417	0.27
Work rate (m/min)	10	66.6 (21.1)	93.8 (26.4)	27.2 (11.5, 42.9)	0.003	1.24
		**Pre-session**	**Post-session**			
**Feeling state**^**c**^	15	2.4 (0.5)	2.6 (0.4)	0.2 (0.0, 2.6)	0.022	0.66

^a^Mean (standard deviation)

^b^Mean (95% confidence interval)

^c^Responses were scored as follows: “sad” = 1, “neutral” = 2, “happy” = 3

## Discussion

Overall, B2La was feasible to deliver within a school setting for adolescents with disability and was moderately well received by students and teachers. Program implementation was high over the course of the intervention, with no adverse events reported. Classroom teachers made several adaptations to HIIT sessions which were noted in our session observations. Improvements in muscular fitness performance and functional performance were observed following the intervention, with students reporting improved mood following participation in HIIT.

Despite increasing interest in school-based HIIT research, data examining adolescents’ enjoyment in HIIT programs is lacking [20]. Overall, the B2La program was mostly enjoyed by students, as evidenced by relatively high levels of program satisfaction. Importantly, teachers also expressed their overall satisfaction with the program, which is likely to have implications for ongoing program delivery. Of note, the majority of students (80%) expressed their intention to continue participating in HIIT, with teachers also willing to continue to deliver the program to future student cohorts. This is an important finding as our study involved limited researcher involvement in terms of session delivery, therefore this approach is likely to be more sustainable and scalable. However, it should be acknowledged that classroom teachers’ satisfaction with program resources (i.e., heart rate monitors and iPad application) was lower than anticipated. While introducing technology can help improve program engagement, it may also add complexity to intervention delivery. Researchers should therefore carefully consider the potential benefit of including technology in their intervention design, against the additional complexity and time requirements.

A key objective of our study was to examine how our existing B2L intervention could be adapted to meet the needs of adolescents with disability. As the B2L intervention was originally designed for adolescents without disability, it was encouraging to see that the program could be adapted in a number of ways to meet the needs of students with disability. First, the B2La sessions typically involved 14–16 students, with four teachers (including learning and support teachers) facilitating session delivery. This resulted in a teacher to student ratio of ~ 1:4, which is much higher than what was involved in our original studies (~ 1:15) [24, 32]. This was considered necessary as previous research has shown that a high instructor-participant ratio is critical to ensure adolescents with disability remain focused and are provided with the individualized instruction and support to participate in physical activity [33]. Indeed, previous exercise interventions for adolescents with intellectual disability have been conducted under highly controlled conditions and have utilized instructor-participant ratios as high as 1:1 [34]. This level of supervision may not always be possible in interventions delivered in schools; it is therefore promising to observe that B2La could be delivered with an instructor-participant ratio of ~ 1:4. However, while an instructor-participant ratio of 1:4 is feasible in NSW schools, this may not be the case for other national or international schools and should therefore be interpreted with caution.

Another adaptation of the B2L intervention involved simplifying HIIT exercises to reduce students’ cognitive demand during the early phase of the intervention. For example, only one exercise was repeated throughout the work interval (i.e., performing squats for 30 s). This allowed for teachers to provide skill-specific feedback to students and help encourage mastery of tasks. As competency developed, teachers encouraged students to complete exercises that required higher levels of complexity (e.g., performing a combination of aerobic and resistance exercises during work intervals). A similar approach was applied in a recent fundamental movement skills intervention involving children with intellectual and developmental disabilities [35]. In their study, Collins and Staples [35] utilized a stations-based approach to develop several sport specific skills (i.e., basketball dribbling, passing, shooting). Each station had several progressions, which increased in skill complexity (e.g., stationary dribble vs dribbling while moving), allowing students to work toward skill mastery. Although this approach is not unique to individuals with disability, it may be an important strategy to help this population attempt and succeed at new tasks [36].

The success of school-based interventions largely depends upon teachers’ ability to implement programs effectively. Lack of time is one of the most commonly cited barriers to school-based interventions [16]. It is therefore promising to see that teachers reported facilitating the prescribed weekly dose of HIIT for the duration of the study period (at least two sessions per week). Importantly, the overall time commitment required to facilitate sessions was deemed acceptable by teachers. However, we acknowledge that what is deemed an “acceptable” amount of time, may differ between schools due to the variability in how academic lessons are structured and prioritized. For example, special education schools (also referred to as schools for specific purposes) may have more flexibility in their curriculum, which may potentially alleviate the “time burden” placed on many mainstream schools. Of note, only two previous school-based HIIT programs have been delivered to adolescents with disability, both of which were conducted in special education schools [21, 22].

Despite session intensity being lower than originally prescribed (~ 70% vs 85% HR_max_), the overall volume of exercise was still sufficient to provide meaningful benefits for students, which is consistent with our findings from the B2L cluster RCT [37]. However, it is difficult to compare our findings with previous school-based HIIT programs targeting adolescents with disability, as the level of intensity reached during HIIT sessions has not previously been quantitatively reported. For example, in the Sport-2-Stay-Fit study, students performed HIIT for 8 weeks to increase their level of fitness prior to a 6-month afterschool program [22]. During HIIT sessions, students were encouraged to perform at an “all-out maximum” intensity for 30 s, followed by active rest of 90 or 120 s. As such, the authors suggested that extensive heart rate monitoring during HIIT sessions was considered unnecessary, and therefore did not provide data regarding the intensity reached during HIIT sessions. In an earlier study, adolescents with intellectual disability performed sprint interval training (SIT) (15 s work to 45 s rest) at an intensity equivalent to the ventilatory threshold (as determined by a maximal exercise test) [21]. However, similar to the Sport-2-Stay-Fit study, heart rate was not monitored during exercise sessions and it is therefore difficult to determine the level of exercise intensity reached by participants.

There is an ongoing debate in regards to what is the minimum threshold for HIIT [38]. While some place this lower limit at 85% HR_max_ [20], others suggest that activity below the threshold of 95% HR_max_ should not be considered HIIT. As such, the exercise performed in the current study may be more accurately defined as “vigorous-intensity interval training”. Although there is some overlap between vigorous- and high-intensity exercise thresholds, vigorous-intensity exercise is characterized as falling between 70 and 90% HR_max_ [39]. While it is likely the potency of training programs are reduced as intensity declines, important health benefits can still be achieved by exercising at lower (although still vigorous) intensity, especially for individuals with low levels of physical fitness. This is particularly important in real-world settings where it is more difficult to control exercise intensity compared to laboratory-based research. Therefore, researchers are likely to face a trade-off between rigid intensity protocols (i.e., > 85% HR_max_) and what is considered to be feasible for delivery in real-world settings such as schools.

Based on our observations using the SAAFE checklist [26], teachers delivered the HIIT sessions with moderate-to-high levels of fidelity. Teachers adhered well to the Supportive, Active, Fair, and Enjoyable principles, but generally failed to implement teaching practices that facilitated autonomy. Given the unique study sample, this finding is not surprising. We did not expect teachers to provide students with high levels of choice, but rather sessions would be more teacher-directed to ensure all students were participating, and appropriate supervision and feedback were provided. This is particularly true during the early phase of the intervention, where students were unlikely to be familiar with the nature of HIIT. Despite the lack of autonomy provided during sessions, relatively high satisfaction with exercise sessions was still reported by students.

Given the low levels of physical activity and fitness typically observed among adolescents with disability [6, 7], interventions targeting this group are warranted. Our study findings provide preliminary evidence for the efficacy of HIIT for improving functional performance and muscular fitness in adolescents with disability. However, these findings have limited generalizability due to the small sample size and only involving ambulatory adolescents, therefore future research should be conducted to confirm these findings. Previous research has also demonstrated the potential efficacy of HIIT for adolescents with physical [22, 40] and intellectual disabilities [21]. One study involving adolescents with cerebral palsy (n = 14) observed a 10% increase in VO_2peak_ following a clinical HIIT intervention. During the study, participants performed intervals of 1.5 to 4 min of high-intensity exercise (targeting ≥ 85% HR_max_), by walking or running on a treadmill [40], for a total of 24 sessions. Another study involving participants with mixed physical disability (n = 70), observed significant improvements in anaerobic (*d* = 0.54) and aerobic fitness (*d* = 0.94), after an 8-week HIIT program [22]. The large effects by Zwinkels and colleagues may be attributed to a higher intensity protocol, with participants completing 30-s sprint exercises (i.e., all out maximal efforts), followed by 90 to 120 s of active rest, for a total of 20 to 25 min. Our findings are somewhat consistent with an earlier study involving adolescents with intellectual disability, which observed a significant effect for the total distance covered in a 6-min walk test [21]. However, unlike the current study, no effect was found in the sit-to-stand test. This is likely due to the nature of the intervention which strictly utilized an aerobic cycling protocol. In our study, both upper (e.g., push-ups, triceps dips) and lower body (e.g., bodyweight squats and lunges) resistance exercises were incorporated into the HIIT sessions, which may explain improvements in both upper and lower body muscular endurance performance. Although improvements in performance were observed, our findigns should be interpreted with caution as we did not include a control group

Finally, we observed a consistent positive effect on students’ mood following participation in HIIT. It is important to note that students’ perception of mood was evaluated using an adapted version of the Feeling State questionnaire, which has not been validated. Although promising, this finding should be interpreted with caution. Our previous research conducted with adolescents without disability has also observed similar post-exercise effects [32, 41]. Although not measured in the current study, we previously found HIIT performed at the start of academic lessons improved students’ on-task behavior [32]. Indeed, the acute affective response of HIIT may act as a mechanism responsible for improvements in academic performance [42]. As such, improved affective responses following HIIT may help create favorable learning conditions by improving goal-directed behaviors and engagement in academic lessons. Such outcomes are likely to be salient for teachers and school executives, which may help the ongoing implementation of physical activity interventions in schools. Therefore, future research examining the potential behavioral and cognitive effects of exercise in this population is warranted. Further, affective feeling state of adolescents with disability using a more robust and validated measure should be assessed.

### Strengths and limitations

The strengths of our study include the unique study population, potential scalable intervention design, and evaluation of multiple aspects of feasibility. However, there are several limitations that should be acknowledged. First, the small sample size and the fact participants were recruited from only one secondary school limits the generalizability of our findings. Second, only ambulatory adolescents were involved in our study; therefore, we were unable to assess the feasibility of the B2La program in non-ambulatory adolescents. Future physical activity interventions targeting youth with disability should include exercises that can be performed by adolescents with physical disability (e.g., boxing exercises, upper body exercises using wrist weights or elastic bands, wheelchair shuttles, body twists, arm swing/rotations). As this was a pilot feasibility study, we did not include a control group and therefore preliminary efficacy findings should be interpreted with caution. Further, due to the impact of the COVID-19 pandemic, researchers were unable to conduct fitness assessments at the study school. As such, teachers were responsible for conducting both baseline and post-test assessments, under the guidance of the research team. Although the 6-min walk/run test demonstrates sufficient feasibility, reliability, and validity in adolescents with disability, limited data is available for the sit-to-stand and push-up tests, limiting the strength of muscular fitness findings. Feasibility data presented from learning and support teachers should be interpreted with caution as these individuals were not responsible for delivering sessions. Their perceptions of the program were collected as an adjunct to those gathered from the classroom teachers and therefore should be considered from a support perspective, rather than a delivery perspective. Finally, retention at post-test was lower than anticipated (< 70%); however, several students were unavailable to perform post-test assessments due to multiple absences from school.

## Conclusion

Schools have an important role to play in the promotion of physical activity among adolescents. Despite their potential, there are few interventions specifically targeting adolescents with disability in school settings. Based on data gathered from this study, we found the B2La program can be delivered to students with disability in a school setting. However, we acknowledge that future research is needed to confirm these findings. Encouragingly, the program was well received by students and teachers. The program resulted in reasonably high intervention fidelity, which is encouraging considering the research team had limited involvement. Preliminary efficacy findings also highlight the potential of HIIT for improving functional performance in adolescents with disability, however, should be interpreted with caution due to the small sample size. A large-scale effectiveness evaluation of B2La in students with disability is warranted.

## Data Availability

The datasets used and/or analyzed during the current study are available from the corresponding author on reasonable request.
